# Cryovacuum setup for optical studies of astrophysical ice

**DOI:** 10.1038/s41598-023-48541-3

**Published:** 2023-11-30

**Authors:** Oleg Golikov, Darkhan Yerezhep, Aigerim Akylbayeva, Dmitriy Yurievich Sokolov, Eugeniy Korshikov, Assel Nurmukan, Abdurakhman Aldiyarov

**Affiliations:** 1 Al-Farabi Kazakh National University, Al-Farabi Av., 71, 050040 Almaty, Kazakhstan; 2 Satbayev University, Satbaev str., 22, 050040 Almaty, Kazakhstan; 3 Institute of Physics and Technology, Satbayev University, Ibragimov str., 11, 050032 Almaty, Kazakhstan; 4 Almaty Technological University, Tole Bi av., 100, 050012 Almaty, Kazakhstan

**Keywords:** Astronomy and astrophysics, Infrared spectroscopy, Phase transitions and critical phenomena, Surfaces, interfaces and thin films

## Abstract

This paper presents a cryovacuum setup for the study of substances under near-space conditions. The setup makes it possible to study the infrared spectra, refractive index, and density of substances that are condensed from the vapor phase onto a cooled substrate in the temperature range from 11 to 300 K. At the same time, it is possible to obtain the ultimate pressure of 1 × 10^–10^ Torr in the vacuum chamber. The presented setup is based on FTIR spectroscopy (the spectral measurement range is 400–7800 cm^–1^) and laser interference, through which the important physical and optical parameters are determined. A number of experiments allow us to point out that the data obtained using this setup correlate well with the experiments of other authors. Due to the non-directional deposition of substances from the vapor phase, the ice formed resembles the one formed under cosmic conditions as closely as possible, which makes the presented setup particularly valuable. The presented cryovacuum setup can be used for the interpretation of data obtained during astrophysical observations, providing a means to determine the properties of cosmic objects.

## Introduction

In recent years, many researchers have focused their attention on studying interstellar dust and astrophysical ices^1–5^. These objects are of particular interest as they are key components of the interstellar medium and play an important role in the formation processes of stars and planets^6–8^. Astrophysical ices are ice formations that form on the surface of microscopic dust particles. These ices consist of various molecules, such as water, methane, ammonia, carbon dioxide, etc.^8–11^. According to astronomical theory, when ice and dust clouds are massive enough, they can collapse and form one or more stars, and the remaining material forms planets and smaller bodies^12,13^. Thus, the interstellar medium is the original matter from which the Earth and many other planets were formed, while comets are remnants of the space that gave birth to them.

Interstellar molecular clouds are quite interesting. They exist at a temperature of about 10 K and have a density of about 10^3^–10^4^ atoms/cm^3^. Such clouds usually exist for about 10^7^ years^14^. Inside these molecular clouds, there are amorphous silicates and dust particles about 0.1 µm in size^8^. These dust particles are so cold that other atoms condense on their surface, causing the formation of an icy mantle. Because of all these processes, reactions occur in the solid phase of the interstellar medium. These reactions cannot exist under normal conditions due to low molecular density. Subsequently, due to ionizing radiation and cosmic rays, the physical and chemical properties of interstellar and cometary ices begin to change. These changes lead to the formation of new molecules and the destruction of the old ones^11,15–17^. As a result of these processes, more complex molecules, for instance, ethanol and methanol, are formed^18,19^.

The data from laboratory studies are still used for the analysis of astronomical spectra of interstellar and planetary ices in order to determine their molecular composition^20–23^. The reference spectra used for these purposes must be unambiguously defined in terms of their composition, the temperature at which they are obtained, their structural phase, and the history of their structural phase transitions. That is why laboratory studies are important to obtain the most accurate data.

To study interstellar dust and astrophysical ices, special laboratory equipment is needed^8,11,17,24,25^. In particular, such facilities make it possible to study the behavior of molecules of substances at very low temperatures, which is of particular importance for studying astrophysical ices.

The use of infrared (IR) spectroscopy is driven by its usefulness as an analytical method that does not require sample modification and its relative simplicity. Scientists have demonstrated the high efficiency of the IR spectra for tracking the structural phase changes of substance molecules in noble gas matrices^26–28^.

This manuscript describes a universal cryogenic setup used to study the optical and physical properties of thin films of a variety of substances under near-space conditions. The availability of such a setup will make it possible to better understand the nature of the behavior of compounds that make up interstellar dust, astrophysical ice, and some other celestial bodies. Testing and comparing the results with those presented in other studies will make it possible to determine the degree of accuracy of the research carried out using the setup and discuss its prospects.

## Materials and methods

**Figure 1:**
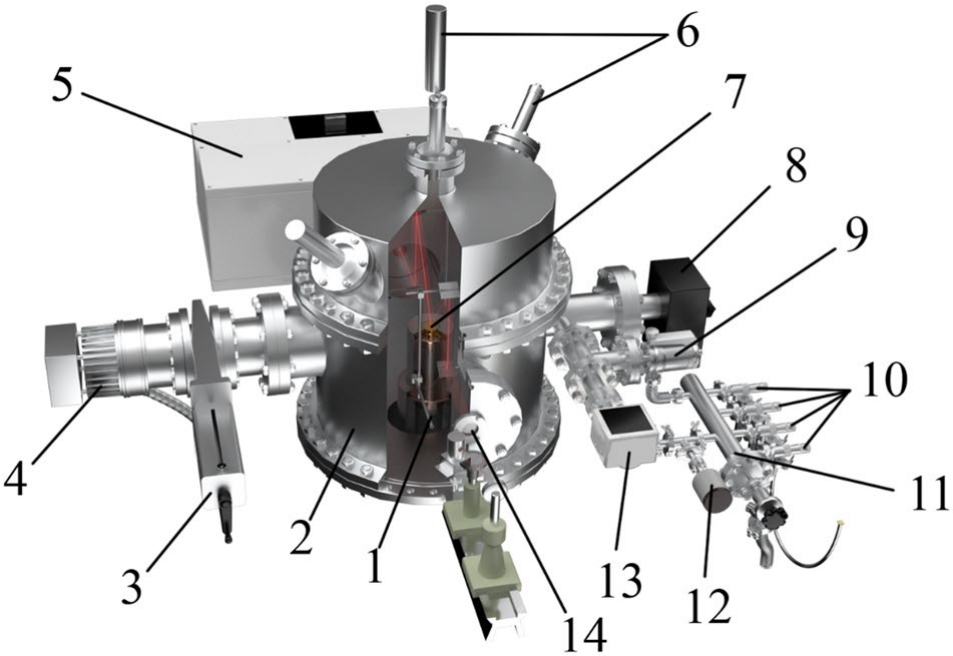
A three-dimensional model of the experimental setup: 1–refrigerator; 2–vacuum chamber; 3–sliding vane pump; 4–turbomolecular pump; 5–FTIR; 6–double beam laser interferometer; 7–substrate; 8–mass spectrometer; 9–high-precision leak valve; 10–needle leak valve; 11–sample preparation system; 12–baratron; 13–pressure sensor; 14–KBr window.

In order to simulate near-space conditions, it is necessary to construct a setup capable of creating a deep vacuum and reaching low temperatures. It is clear that for this purpose, the setup needs air extraction systems and a system used for cooling to low temperatures. In this case, since the setup is to study strictly defined substances, a system for precise control of the composition of the mixture under study and a mechanism for controlling the size (thickness) of the compound under study are necessary. We have developed a setup that meets these requirements. It is presented in three-dimensional form in Fig. 1.

**Figure 2:**
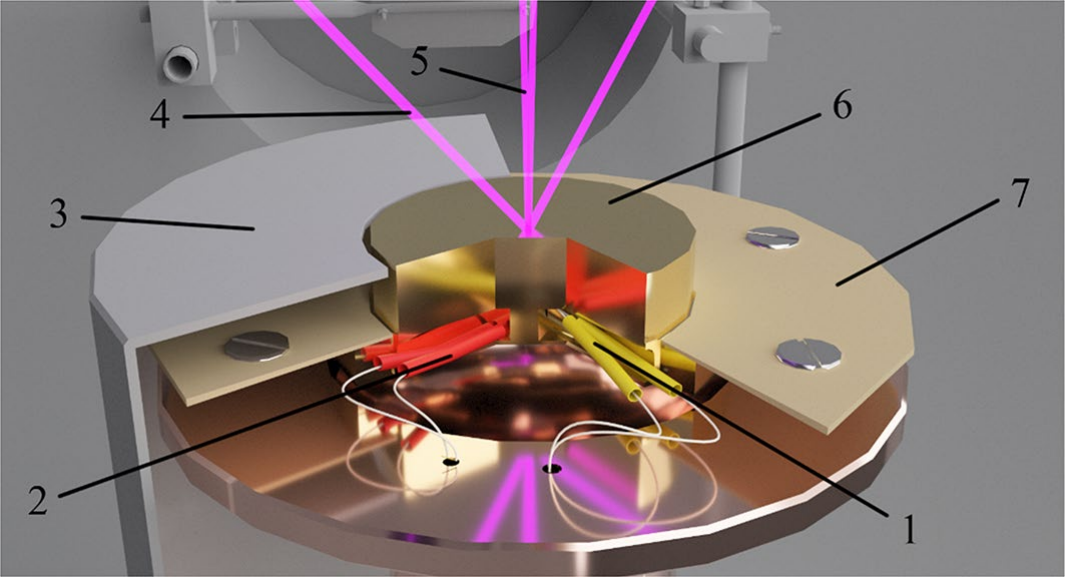
A three-dimensional model of a part of the setup near which the substrate is located: 1–heating element; 2–thermal sensor; 3–protective cover; 4–laser α_2_ = 45°; 5–laser α_1_ = 1°; 6–gold-coated substrate; 7–pressure ring.

The setup is able to reach the low-temperature region using a Gifford–McMahon micro cryogenic machine (Fig. 1, 1), which is capable of operating at temperatures starting from 11 K. The machine is located in the center of a vacuum chamber in the form of a cylinder (Fig. 1, 2) made of stainless steel with a radius of 215 mm and a height of 420 mm. In order to allow IR and laser emission to pass through the chamber, optically transparent KBr windows were installed on several sides.

**Figure 3:**
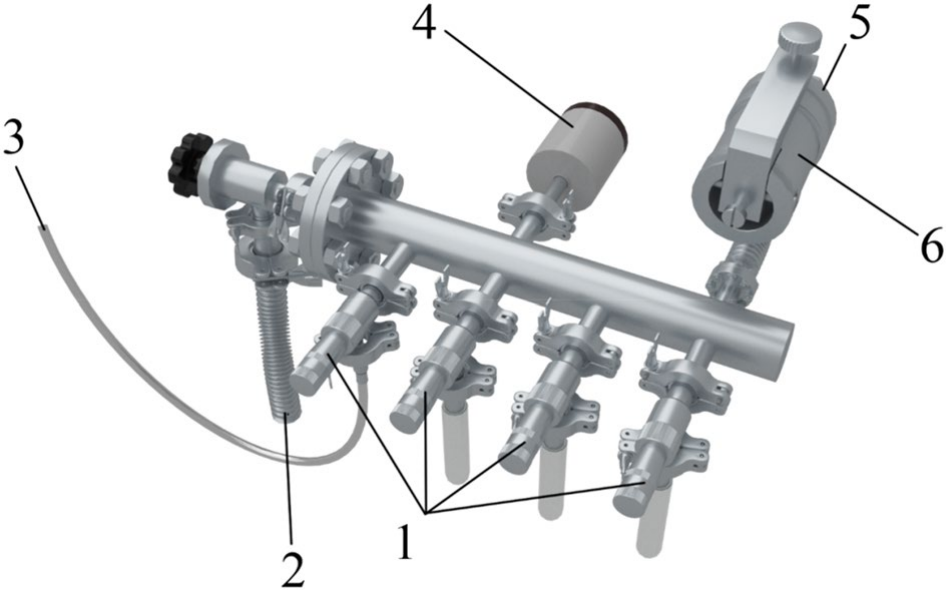
A three-dimensional model of a part of the setup used to create the mixture under study: 1–needle leak valves; 2–corrugated cable leading to the fore vacuum pump; 3–gas supply hose; 4–pressure sensor; 5–route to the vacuum chamber; 6–high-precision leak valve.

**Figure 4:**
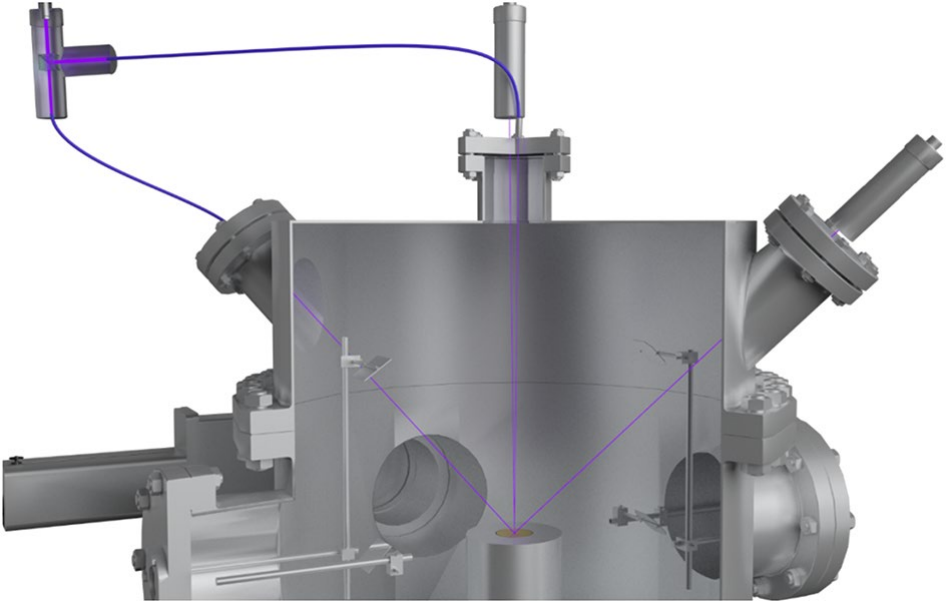
A three-dimensional model of the setup in sectional view showing the incidence of the laser beams on the substrate.

The vacuum generation system includes a Turbo-V-301 turbomolecular pump (Fig. 1, 4) (Agilent, USA) that works together with an SH spiral fore vacuum pump (not shown in the figure) (Agilent, USA). The vacuum system is connected to the vacuum chamber through a CFF-100 sliding vane pump (Fig. 1, 3). Such a system is capable of obtaining oil-free, pure vacuum up to P = 1 × 10^–10^ Torr. The vacuum is measured using a CC-10 vacuum gauge (Televac, USA) (Fig. 1, 13). An XT100 quadrupole mass spectrometer (Fig. 1, 8) (Extorr, USA) is used to analyze the composition of the mixture in the vacuum chamber.

**Figure 5:**
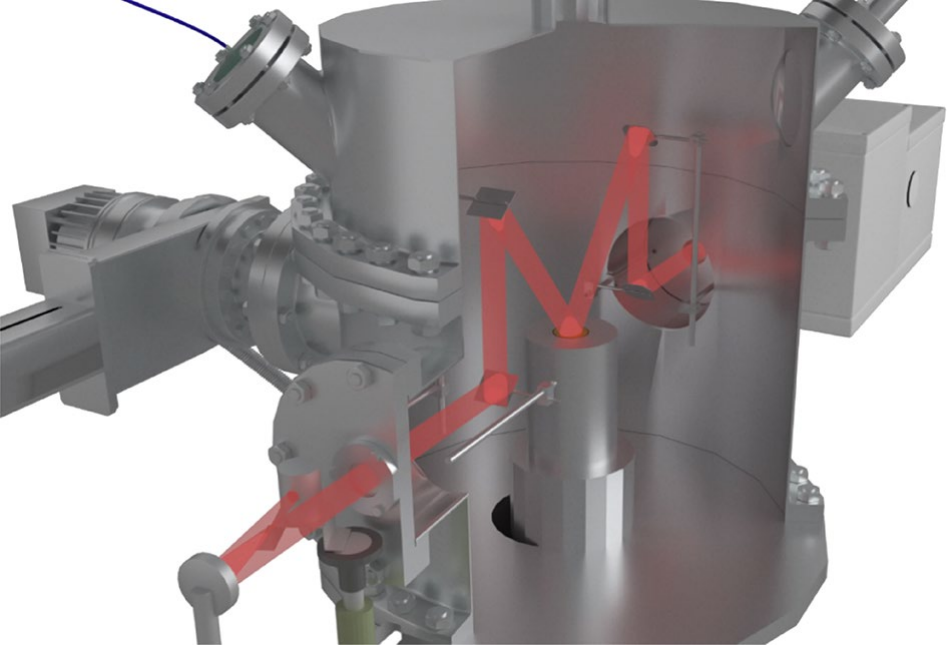
A three-dimensional model of the vacuum setup in sectional view showing the course of the IR beams inside it.

A copper substrate (Fig. 1, 7) (purity: 99.99%) coated with a thin layer of gold (purity: 99.99%) is located on top of the micro cryogenic machine. Before coating the substrate with a layer of gold, we carefully polished it to achieve a roughness of *Ra* = 0.012. An approximate 3D model of the copper substrate in sectional view is shown in Fig. 2. Both the heating element (Fig. 2, 1) and the temperature sensor (Fig. 2, 2) reach the center of the substrate (Fig. 2, 4) through special channels for more consistent heating and temperature readings. A special casing (Fig. 2, 3) protects the internal part from the vapor condensing on the substrate and filling the vacuum chamber. The pressure ring (Fig. 2, 5) is necessary for good surface contact between the substrate and the upper flange of the refrigerator. Good surface contact eventually provides good heat transmission from the refrigerator. The gasket between the substrate and the flange is an indium gasket that remains soft at low temperatures and has sufficient thermal conductivity.

Temperature measurement and heating of the substrate and the substance under study are performed using a DT 670 semiconductor temperature sensor controlled by an LS325 thermal controller (LakeShore, USA). The interface of this thermal controller was transferred to the LabView software for convenience, which made it possible to reach and stabilize the temperatures required for the study faster. The data received from the ADC L-Card E20-10 and the LS325 thermal controller are displayed in the form of a self-recording device. For convenience, the temperature of the substrate on which the substance under study is deposited is also displayed in degrees Kelvin in a separate field in a numerical form.

**Figure 6:**
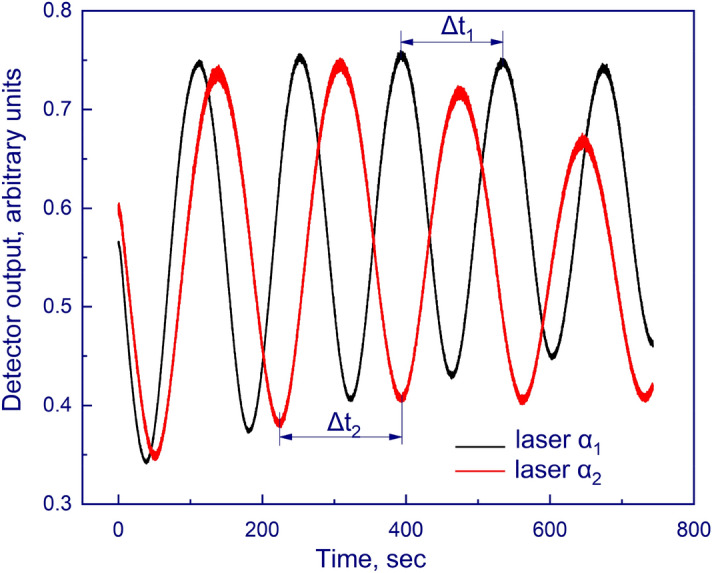
Graphs of interferograms of the two lasers inside the vacuum setup.

An approximate 3D model of the sample preparation system is shown in Fig. 3. The sample preparation system (Fig. 1, 11; close-up Fig. 3) consists of four GW-J needle leak valves (Fig. 1, 10; Fig. 3, 1) and a 951–5106 high-precision leak valve (Fig. 1, 9; Fig. 3, 6) (Agilent, USA). Needle leak valves are used to inject the substances into the sample preparation system, where mixtures with the required concentrations are prepared if necessary. High-precision leaks can be used to inject the material from the sample preparation system directly into the vacuum chamber.

The system has an additional SH vacuum pump used to clean the sample preparation chamber before creating the mixture under study (Fig. 3, 2). The pressure in the sample preparation system is controlled by a baratron (Fig. 1, 12; Fig. 3, 4) that works with a PR 4000 interface (MKS Instruments, USA). The sample preparation system is connected to the vacuum chamber via a high-precision leak valve (Fig. 3, 6).

A double beam laser interferometer (Fig. 1, 6) consisting of a laser (wavelength λ = 405 nm) and two P25A photoelectronic multipliers (PMTs) (Sens-Tech, UK) with maximum sensitivity at λ = 400 nm makes it possible to determine the growth rate of the substance under study along with its thickness, density, and refractive index based on the interference pattern. A beamsplitter cube with 50% reflection and 50% transmittance is used to receive two laser beams from a single source. The beams received and focused at the end of the light guides fall on the substrate at the angles α_1_ = 1° and α_2_ = 45° (Figs. 2, 4 and 5). Figure 4 below shows a 3D model displaying the laser beams inside the vacuum chamber and their incidence on the substrate.

Optical spectra of the molecular compounds under study are detected using an FSM 2203 FTIR spectrometer (Fig. 1, 5). This spectrometer operates in the infrared spectral range (370–7800 cm^–1^) and has a maximum spectral resolution of 0.125 cm^–1^. The path of the infrared rays is shown in Fig. 5.

Before the beam is detected by the IR spectrometer, the IR beam from the radiation source passes through a system of 5 mirrors. The first mirror is concave and is capable of collecting light. It is necessary to form a focused IR beam. After that, the IR beam passes through a potassium bromide glass, which is transparent for the given wavelength range. Next, the beam enters the substrate through a system of two mirrors, is reflected by the substance under study, passes through the two mirrors, and exits through the potassium bromide glass on the other side, reaching the Fourier-transform infrared spectrometer. The last mirror in the system is a scattering mirror.

The experiment consists of the following stages:

*Stage 1*: Before starting the experiment, it is necessary to clean the vacuum chamber volume to a pressure of 1 × 10^–10^ Torr, which is close to the space vacuum condition. For this purpose, the turbomolecular pump and the fore vacuum pump are used together. Creating a vacuum in the research chamber takes about 3 h on average. It is important to note that the chamber almost constantly maintains a pressure of 1 × 10^–6^–1 × 10^–7^ Torr. As a result, it takes less time to reach a deeper vacuum state than it would have otherwise.

*Stage 2*: The Gifford–McMahon machine is activated to reach the low-temperature region on the substrate on which the substance under study will be deposited. It takes 50 min to reach an operating temperature of 11 K. After that, the setup is ready for the deposition of the substance under study.

*Stage 3*: The creation of the test substance in the sample preparation system is carried out simultaneously with the cooling of the substrate by the refrigeration unit. The volume into which the generated mixture is injected is pre-pumped with a fore vacuum pump to create a clean environment. With the help of the needle leak valves, the required substance or substances, if the compound to be tested is a mixture, are injected into the prepared volume. A mass spectrometer is used if the purity of the generated mixture has to be further verified.

*Stage 4*: Once the operating temperature has been reached and the creation of the mixture under study has been completed, the IR background spectrum is obtained. After that, the high-precision leak valve is opened, and the test substance enters the vacuum chamber. In the vacuum chamber, the substance is condensed from the vapor phase (PVD) onto the cooled substrate. Background injection, in contrast to guided injection, makes it possible to obtain thin films of uniform thickness over the entire surface of the substrate^29^. The pressure at a given operating point is monitored to ensure uniform adhesion of the substance and its deposition on the substrate. Control of the growth of the deposited film on the substrate surface is performed using an interferogram obtained from two photomultipliers connected to an ADC.

*Stage 5*: Once the film stops growing and reaches the required thickness, the volume of the vacuum chamber is pumped out again to get rid of residual vapors. Afterward, a series of IR spectra are obtained at the required temperature points. Temperature stabilization at these points is performed by the LS325 thermal controller, which has been improved using the LabView software^30^.

The data obtained in the course of the experiment make it possible to calculate important optical parameters using indirect methods.

### Refractive index

Many researchers use the refractive index as a characteristic that helps them find out more about the structure of a substance and identify other substances. The reason for this is that the refractive index is one of the fundamental properties of a substance when studying it by means of electromagnetic waves^31^. The determination of the refractive index of the substance under study is performed according to the interference picture obtained from the PMT. Figure 6 shows the interference pattern of one of the experiments in the form of two interferograms for a laser incident at an angle α_1_ = 1° and a laser incident at an angle α_2_ = 45°.

The film thickness *d* was determined for any interference maxima or minima according to the following equations:

$${d}_{max}=\frac{\lambda m}{2\sqrt{{n}^{2}-{sin}^{2}\mathrm{\alpha }}},$$

where *m* = 1, 2, 3, … is the order of interference for the maxima, and

$${d}_{min}=\frac{\lambda (m+0.5)}{2\sqrt{{n}^{2}-{sin}^{2}\mathrm{\alpha }}},$$

where *m* = 0, 1, 2, 3, … is the order of interference for the minima.

The growth rate is determined by dividing the thickness of the film by the time *t* during which it was deposited on the substrate. Δt_2_.

The refractive index n is calculated using the double-angle method, as described in Ref.^32^:

1 $$n=\sqrt{\frac{{\Delta t}_{2}^{2}{\mathrm{sin}}^{2}{\mathrm{\alpha }}_{2}{-\Delta t}_{1}^{2}{\mathrm{sin}}^{2}{\mathrm{\alpha }}_{1}}{{\Delta t}_{2}^{2} {- \Delta t}_{1}^{2}},}$$

where Δ*t*_*1*_ and Δ*t*_*2*_ are the periods of interference minima and maxima for the angles of incidence α_1_ and α_2_, respectively.

The use of formula (1) is appropriate when the deposition rate of the substance is constant, that is, when the periods of the interference bands are constant.

### Density

A number of researchers calculate the density of substances in their works using the value of the refractive index they obtain^33–35^. The relationship between the refractive index and density is mathematically represented in the form of the Lorentz-Lorenz equation^36^:

2 $$\frac{{n}^{2}-1}{{n}^{2}+2}=L\cdot \rho ,$$

where *L* is the Lorentz-Lorenz coefficient or factor in cm^3^/g^37^.

Researchers have shown a high degree of consistency between the theoretical data obtained using this formula and the experimental values of the densities of various substances^38,39^. In order to apply the Lorentz-Lorenz equation, the following condition must be fulfilled: the pores in the film structure must be smaller in size than the wavelength of the light to which the film is exposed. In other words, the film must be homogeneous for the laser wavelength so that the scattering of light on inhomogeneities of the film is negligibly small.

If the relation (2) is valid, then one can similarly calculate^40^ the numerical value of the Lorentz-Lorenz factor *L* for other substances using the values of *n* and *ρ* from the experiments or literature.

## Results and discussion

The FTIR spectrometer has to be calibrated before researching low-temperature thin films using vibrational spectroscopy in the temperature range of 15–150 K. Spectral sensitivity calibration is important for eliminating systematic errors that occur due to different external and internal factors.

The uncertainty of wavenumber graduation is determined by direct measurement as the difference between the wavenumber value of a standard sample (polystyrene film, 30 nm thickness) along the abscissa and the actual value of the wavenumber obtained from the control record in the IR Fourier spectrometer passport. The measurement uncertainty characteristics are calculated in accordance with the following mathematical model:

3 $$\Delta v= {v}_{i}-{v}_{ref}+{\delta }_{S1}+{\delta }_{S2}+{\delta }_{n.c.},$$

where ${v}_{i}$ is a wavenumber value corresponding to the average ordinate of the absorption line recorded on the calibrated spectrophotometer, cm^–1^; ${v}_{ref}$ is an actual wavenumber value corresponding to the same line, indicated in the control record of the passport of the FTIR spectrometer, cm^–1^; ${\delta }_{S1}$ is a correction for the uncertainty component of the standard sample, cm^–1^; ${\delta }_{S2}$ is a root-mean-square deviation from scale linearity, cm^–1^; ${\delta }_{n.c.}$ is a correction for errors resulting from additional parameters that are unaccounted for, cm^–1^.

According to the Guide to the Expression of Uncertainty in Measurement^41^, uncertainty sources can be grouped into two categories: type A (uncertainty estimation by means of a statistical analysis of observation series) and type B (uncertainty estimation by means other than a statistical analysis of observation series). Afterward, the analysis of uncertainties in input quantities and correlations was carried out (Table 1). As a result of the analysis of formula (3), the components of uncertainty were determined. Input quantities are treated as uncorrelated. The influence coefficients (sensitivity) of all variables are equal to 1.

**Table 1:** Uncertainty budget.

№	Input value	Uncertainty type	Distribution	Standard uncertainty, cm^–1^	Sensitivity coefficient	Uncertainty contribution, cm^–1^
1	${v}_{i}$	B	rect	–	–	–
2	${v}_{ref}$	B	rect	0.0170	1	0.1700
3	${\delta }_{S1}$	A	normal	0.0800	1	0.0800
4	${\delta }_{S2}$	A	normal	0.0010	1	0.0010
				0.0010		0.0010
				0.0010		0.0010
				0.0004		0.0004
				0.0010		0.0010
				0.0030		0.0030
5	${\delta }_{n.c.}$	B	rect	0.0140	1	0.1400
Total standard uncertainty						0.4800
Expanded uncertainty						0.9500

In order to verify the convergence of the measurement results, experiments were carried out and compared with the astrophysical studies of other researchers. It is known that condensed molecules of H_2_O and CO_2_ are the most common components of astrophysical ice. In this connection, we chose H_2_O and CO_2_ as the main substances. In the experiments, we used CO_2_ of 99.999% purity (ISKHAN TEHNO-GAS LLP, Almaty, Kazakhstan) with a maximum oxygen volume fraction not exceeding 0.0005%, water vapor not exceeding 0.0007%, and distilled water with a mass concentration of residue after evaporation not exceeding 0.005% of volume.

**Figure 7:**
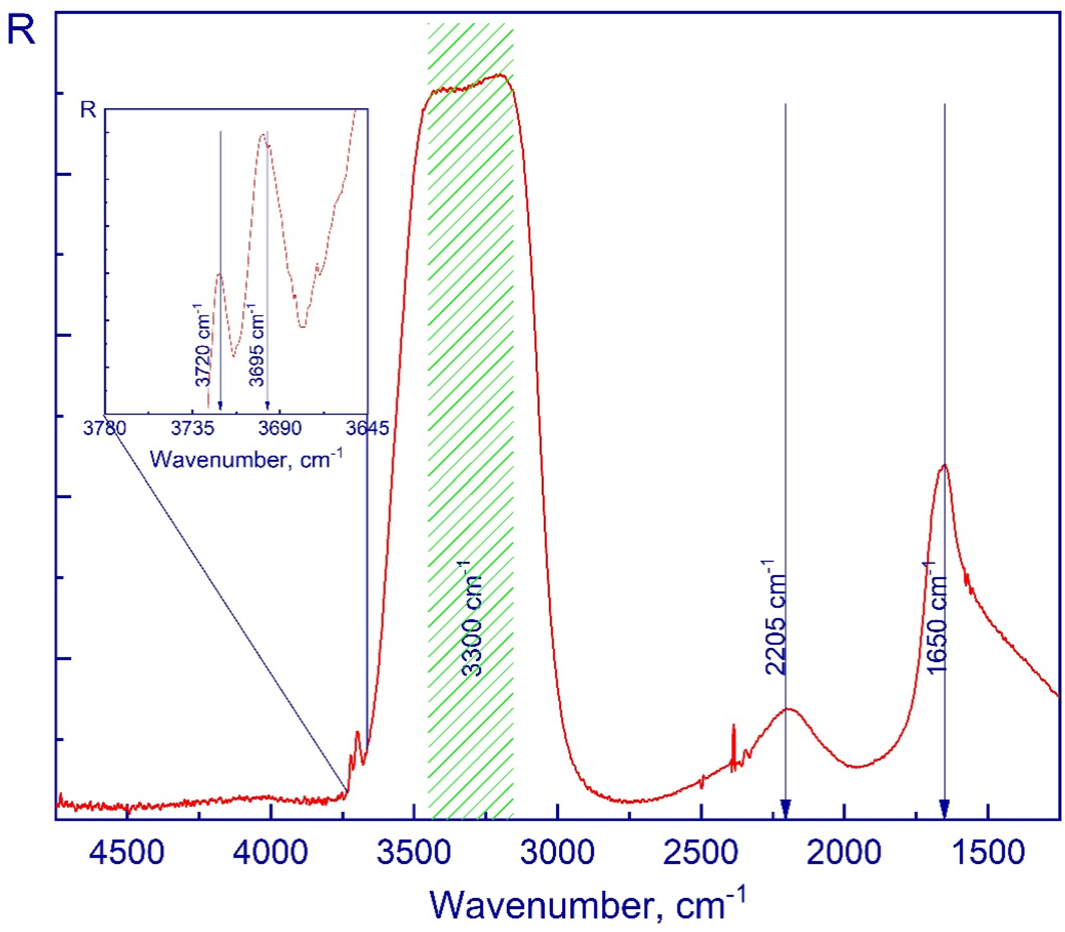
Mid-IR spectrum of an amorphous water ice film deposited at 15 K.

Figure 7 shows molecular spectra of a porous amorphous structure of water (H_2_O) at 15 K obtained in the mid-infrared range. The structure was obtained by vapor phase deposition (PVD) in the described cryovacuum setup. The main absorption bands of porous amorphous solid water are the spectra at 3300 cm^–1^ (symmetric and anti-symmetric stretching modes), 2205 cm^–1^ (combination mode), and 1650 cm^–1^ (overtone of the libration mode and H–O–H bending mode).

As other authors noticed^1,42,43^, also in our study there is a peculiarity (see the inset to the figure) that stems from micropores in the amorphous structure, visible in the large wavenumbers range of the large wavenumbers of the bands of the O–H valence modes: two peaks at 3720 cm^–1^ and 3695 cm^–1^ due to the two- and three-coordinated water molecules, respectively.

**Figure 8:**
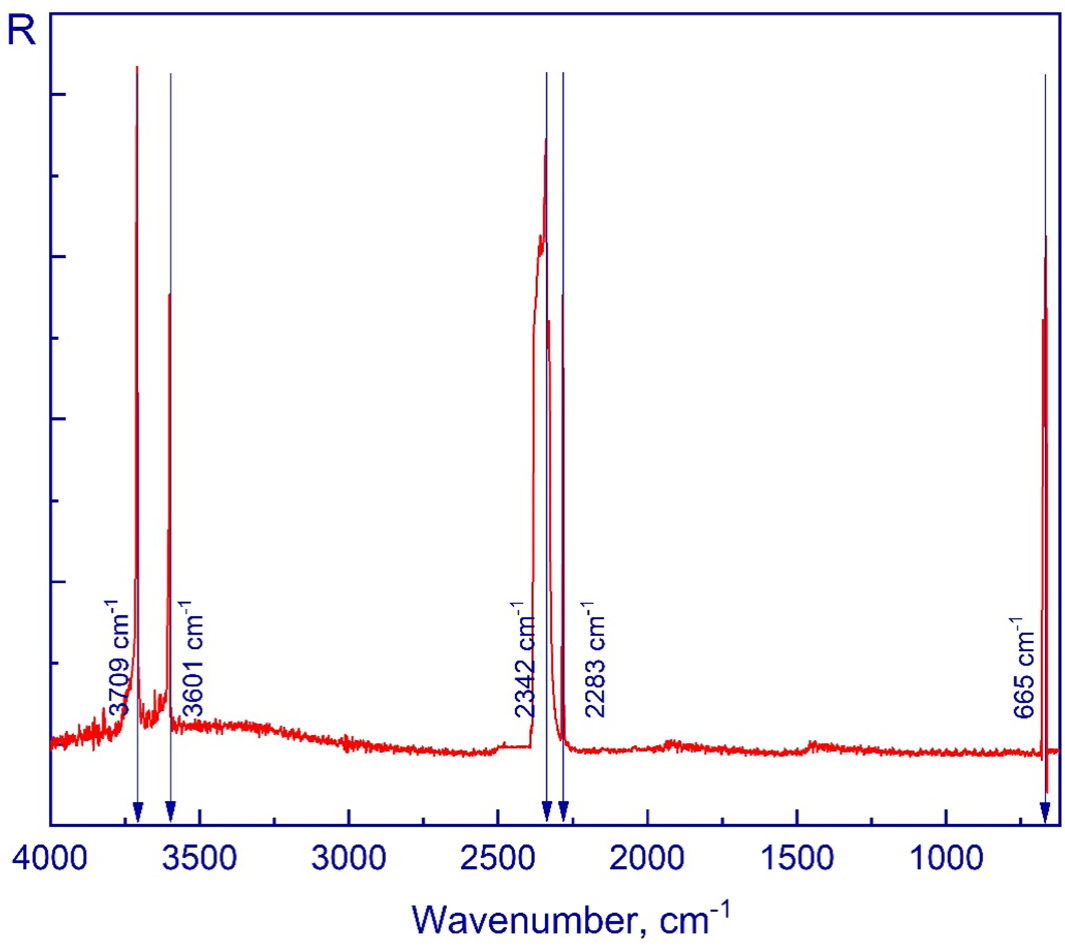
A spectrum of a solid CO_2_ film deposited at 15 K in the mid-IR range.

Figure 8 shows the mid-infrared spectrum of a CO_2_ film deposited and obtained in the described cryovacuum setup at 15 K. Five characteristic bands are highlighted: 3709 cm^–1^, 3601 cm^–1^, 2342 cm^–1^, 2283 cm^–1^, and 655 cm^–1^. These bands can be observed in the works of other authors^10,15,44^.

For convenience, the data obtained are presented in Table 2.

**Table 2:** Main vibrational peaks of H_2_O and CO_2_.

Substances	Our studies		Works of other authors	
	Temperature, K	Peak positions, cm^–1^	Temperature, K	Peak positions, cm^–1^
H_2_O	15	3720 ± 0.95	10–20	3720
		3695 ± 0.95		3695
		3300 ± 0.95		3300
		2205 ± 0.95		2205
		1650 ± 0.95		1650^1,42,43^
CO_2_	15	3709 ± 0.95	10–20	3708
		3601 ± 0.95		3600
		2342 ± 0.95		2343
		2283 ± 0.95		2283
		665 ± 0.95		665^10,44,45^

Using the values of the refractive index and density of solid molecules of H_2_O and CO_2_ provided in Ref.^40^, we calculated the Lorentz-Lorenz coefficient. After that, we used the Lorentz-Lorenz factor for the values of the refractive index *n* we determined and were able to determine the density of the thin films of the cryo-condensates of the studied substances *ρ* at 15 K. The obtained values of the refraction coefficient and density of the substances are presented in Table 3.

**Table 3:** Comparison of the refractive indices and densities.

Composition	Deposition temperature, K	Refractive index	Lorentz-Lorenz coefficient, cm^3^/g	Density, g/cm^3^	References
H_2_O	15	1.20 ± 0.02	0.20	0.620	This work
H_2_O	20	1.20 ± 0.01	0.20	0.640	^46^
H_2_O	20	1.19	0.20	0.600	^47^
H_2_O	22	1.19 ± 0.02	–	–	^39^
CO_2_	15	1.23 ± 0.01	0.13	1.100	This work
CO_2_	14	1.28 ± 0.01	0.14	1.195	^48^
CO_2_	20	1.22	–	–	^46^
CO_2_	25	1.25	0.13	1.100	^38^

According to Ref.^46^, the calculated density of H_2_O equaled 0.64 g/cm^3^, while in our case the deviation did not exceed 5%. In the case of CO_2_ ice, work of Ref.^48^, resulted in ρ = 1.195 g/cm^3^ at a temperature of 14 K. In our case, the difference in density is less than 8%.

## Conclusion

In our study, we presented and discussed the setup of a universal cryogenic spectrophotometer for measuring the physical and optical properties of substances at low temperatures and under deep vacuum conditions, making it possible to facilitate the experimental conditions close to those of open space. This setup allows one to carry out simultaneous measurements of spectral characteristics and interferograms, based on which the refractive index and density of the substances under study are calculated. The temperature at which it is possible to conduct experiments using the setup ranges from 11 to 300 K with a maximum lower pressure threshold of 1 × 10^–10^ Torr. The range of the obtained spectra is 400–7800 cm^–1^. The availability of a sample preparation system makes it possible to study not only pure substances but also impurities in a variety of ratios.

The results obtained with sample substances, H_2_O and CO_2_, demonstrate that the data obtained using our setup correlate with the experimental data provided by other authors. This confirms a sufficiently high experimental accuracy of the studies carried out via this setup.

The results of these measurements correlate with the data provided in other works, as the discrepancies are below the expanded standard uncertainties. Deviation uncertainties below 5% can be reached at a calculated density of H_2_O, and when calculating the density of CO_2_ ice, the deviation was 8%. Thus, the setup presented in this article makes it possible to grow astrophysical ice and carry out the measurements described in the work with certain accuracy. The mass spectrometer makes it possible to perform additional identification of substances inside the chamber. Furthermore, since we plan to focus our future research on studying the effects of ultraviolet radiation on the molecular compounds inside the chamber, the use of a mass spectrometer will be required to identify the compounds obtained.

## Data Availability

The data presented in this study are available on request from the corresponding author.
